# A Rare Case Report: A Malignant Histiocytic Tumor in the Form of Ovarian Mass

**DOI:** 10.1155/2018/1792358

**Published:** 2018-02-11

**Authors:** Emsal Pinar Topdagi Yilmaz, Yakup Kumtepe, Yunus Emre Topdagi, Seray Kaya Topdagi

**Affiliations:** ^1^Department of Gynecology and Obstetrics, Atatürk University School of Medicine, Erzurum, Turkey; ^2^Clinic of Gynecology and Obstetrics, Nene Hatun Gynecology and Obstetrics Hospital, Erzurum, Turkey

## Abstract

Histiocytic cell malignancies are very rare. Hence, the information about this disease in hematology is limited. In this case report, we present a case of malignant histiocytic tumor affecting the ovary of a 40-year-old virgin female. Primary ovarian malignancy was not considered for the patient who was approached as if she had ovarian malignancy, since there was an indication of a mass in the ovary. Therefore, an aggressive surgery was not performed. Since our patient was in the reproductive age, fertility-preserving surgery was performed. Our patient was then treated systemically by medical oncology. In conclusion, the rare malignancy group was investigated in the present study along with an evaluation of the current literature.

## 1. Introduction

Histiocytic and dendritic cell malignancies have been classified as lymphomas, sarcomas, or histiocytic neoplasms, whereas this classification has been currently abandoned by the World Health Organization. Dendritic cell neoplasms were grouped under five subtitles. This classification was constructed as follows: Langerhans cell histiocytosis (LCH), Langerhans cell sarcoma, interdigitating dendritic cell sarcoma, follicular dendritic cell sarcoma/tumor, and other rare dendritic cell tumors (i.e., fibroblastic reticular cell tumor and indeterminate dendritic cell tumor).

Histiocytic sarcomas are malignant proliferation of cells showing morphological and immunophenotypic characteristics of mature tissue histiocytes [1, 2]. Currently, there is no standard protocol for treatment. Depending on the involvement of the disease, surgery, radiotherapy, or systemic chemotherapy may be preferred [3, 4].

The LCH group is a clonal neoplastic proliferation of Langerhans type cells expressing CD1a, langerin, and S100 protein. It is usually diagnosed in childhood. Its annual incidence is approximately one in five million [5]. LCH with an unknown etiology is a group of diseases in which atypical histiocytes cause damage locally or extensively as a result of their accumulation in various tissues such as skin, bone, lung, liver, lymph nodes, mucocutaneous tissues, and endocrine organs [6, 7].

Histiocyte-associated neoplasms are rarely seen in the genital system. It is most commonly seen as LCH in the vulva [8]. Herein, we present a female case of lymphoid pathology in the genital system and discuss its clinical, radiological, and histopathological findings with respect to the literature data.

## 2. Case Report

A 40-year-old patient was admitted to another center with complaints of weight loss and sweating about one year ago before she was referred to our hospital. Multiple benign lymph nodes in the longitudinal, axillary, and inguinal regions were detected by ultrasonography (USG). In the abdominal USG, a pelvic mass with the size of 6 × 7 cm was detected in the neighborhood of left ovary at the posterior of the uterus corpus. Magnetic resonance imaging (MRI) confirmed a mass of approximately 8 × 5 cm in the left ovarian localization. In addition, conglomerated lymph nodes with 4 cm in size were detected in the para-aortic region. The entire body was evaluated as normal on MRI about 12 months ago.

Left salpingo-oophorectomy + para-aortic lymph node dissection + tumoral debulking were performed in the operation. The patient's frozen biopsy result was reported as malignant appearance of unknown primary. Lymph nodes with a conglomerate mass appearance in the para-aortic region were concurrently sent for simultaneous frozen biopsy investigation. As a result, the operation was terminated when it was reported that a lymphoid malignancy might be present. The pathology result was reported as malignant lymphoma. For the pathological investigation of the patient who underwent salpingo-oophorectomy due to suspected ovarian malignancy, in the first health center, it was considered that there was malignant lymphoma or lymphoid pathology, but it was not related to a primary ovarian origin. However, the patient consulted another external center for the definite diagnosis and type identification. None of the immunohistochemical studies performed at the other center helped the diagnosis (CD136, CD10, CD20, CD23, CD3, CD30, CD31, CD68, CD1a, CD34, EMA, Calretinin, DOG1, PAX8, S100, WT1, D2-40, ER, PR, Vimentin, and Pan-Keratin were used as immunohistochemical stains). Dr. Robert Young from Mass General Hospital (Boston, USA) was asked for his opinion, as a definite result was not obtained from the evaluation of the patient's samples. He thought that the neoplasm did not originate from the genital system. Therefore, the case was sent to Dr. Metin Özdemirli from Washington University where immunohistochemical studies revealed that the tumor reacted positively with CD33. The tumor that could not be classified in this center was evaluated as “malignant histiocytic tumor.” Since immunohistochemistry did not help, this diagnosis was made following a process of elimination. After discharge, the patient was referred to a medical oncology clinic.

**Figure 1:**
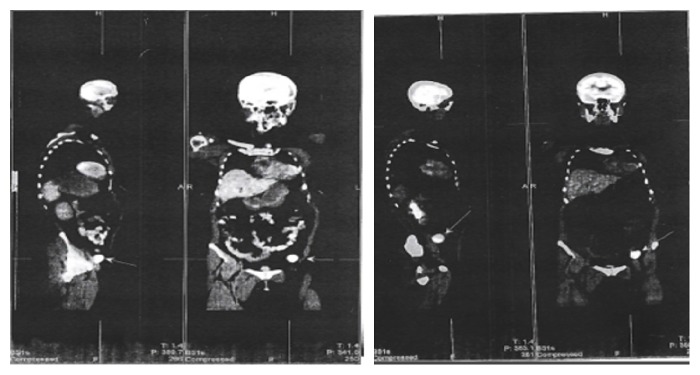
PET-CT images of the patient taken six months apart after recurrence. The place shown with the arrow is overmassed.

**Figure 2:**
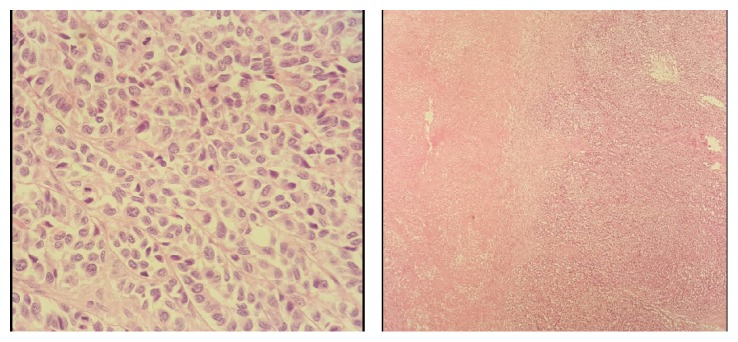
Microscopic images of malignant histiocytic tumor and its necrosis area.

The patient was applied (and admitted) to our clinic due to the recurrence of the mass about one year after the first operation and the subsequent chemotherapy. The patient's hemoglobin value was 7 mg/dL. Other laboratory test results were at normal levels. Two F-18 fludeoxyglucose (FDG) positron emission tomography images taken at four months intervals showed intense hypermetabolic lymph node involvement and an increase in FDG metabolism in the left bowel localization of the abdomen, in the left external iliac area, in the left lumbar, and in the left paravertebral area (Figure 1). During surgery, about 4-cm masses located between the psoas muscle and the external iliac artery under the left round ligament and 6-cm masses with a fixed-appearance and irregularly margins originating from the pelvic region and extending to the location of the left renal vein (including the iliac artery bifurcation and the left ureter also) were excised. In the pathological investigation of the materials obtained from the later operation regarding the recurrence of the mass, a tumoral structure, separated by collagen bundles exhibiting a nodular distribution pattern in the sections, was observed. Additionally, tumor-forming cells were observed as 3-4-fold greater in size than lymphocytes with pleomorphic irregular nucleus and large cytoplasm. Moreover, microscopic investigation displayed that the nuclei were occasionally cleaved, with clear cell changes in some areas, and there were seldom giant cells. Disseminated geographic necrotic areas were detected and three or four mitoses were observed at ten times magnification (Figure 2). In immunohistochemical studies, Vimentin, S-100, CD1a, HMB 45, CD34, CD43, CD13, CD23, CD21, CD117, and MPO were detected to be negative. CD45 was positive in most of the reactive lymphocytes, whereas CD3 was positive in all reactive T lymphocytes. On the other hand, no expression was detected in tumoral cells. Although Pan CK and EMA were weakly positive focally, it was not found to be significant. Focal weak staining was detected after the inhibin treatment. The Ki-67 proliferation index was 15%–20%. This tumor could not be classified by the present immunohistochemical data applied by the pathologists. The patient's report for the recurrence pathology also included details supporting the old diagnosis. The patient was discharged from the clinic with full recovery and referred to the medical oncology service.

## 3. Discussion

Langerhans cells are peripheral dendritic antigen processing cells of bone marrow origin. They are found throughout the body. They play an important role in localized immune response to antigens [9]. Histiocytic and dendritic cell malignancies have been classified as lymphomas, sarcomas, or histiocytic neoplasms, whereas this classification has been currently abandoned by the World Health Organization. Dendritic cell neoplasms were grouped under five subtitles, and histiocytic sarcomas are also one of these groups. Histiocytic sarcomas are malignant proliferation of cells showing morphological and immunophenotypic characteristics of mature tissue histiocytes [1, 2].

The Histiocyte Society classifies diseases as Single System or Multisystem depending on the extent and localization of the diseases [10]. Although a definite classification was unable to be done in the pathology reports, the involvement of the lymph nodes suggested a systemic involvement in our patient who was thought to have a malignant histiocytic tumor as a pathological diagnosis [1, 2]. Moreover, it was also very rare for obstetricians to detect adnexal masses. To our knowledge of the literature, the aforementioned pathology in ovary has not been reported before. Combination chemotherapy was applied as the treatment for lymphoma [3, 4]. The method of primary treatment was still debated. Surgical resection was performed in our patient both to reduce tumor burden and to clarify the pathologic diagnosis.

The pathogenesis of the disease still remains controversial although two main theories emerge: neoplastic versus reactive processes. The first is supported by the fact that the proliferation of Langerhans cells appears to be monoclonal in nature due to somatic changes in tumor suppressor genes [11]. Whether it is neoplastic or reactive is yet to be proved.

The ADNEX risk model can be used by medical doctors to diagnose ovarian cancer in women who have at least one persistent adnexal (ovarian, para-ovarian, and tubal) tumor and are considered to require surgery [12]. The ADNEX model uses nine predictors: There are three clinical variables as age, serum CA-125 level, and type of center (oncology referral center versus others) and six ultrasound variables as maximal diameter of the lesion, proportion of solid tissue, more than 10 cyst locules, number of papillary projections, acoustic shadows, and ascites. The parameters used in ADNEX are based on the terms and definitions published by the IOTA group. The mass in the ovary we are talking about is malignant according to the IOTA criteria. Based on this, we decided for an operation.

Histiocytic cell malignancies are very rare, and hence the information about this disease in hematology is limited. Primary ovarian malignancy was not considered for the patient who was approached as if she had ovarian malignancy, since there was an indication of the mass in the ovary. Only 18 female genital tract histiocytic cell malignancy cases have been previously reported in medical literature in which all of the reported patients have presented papulous or ulcerative lesions on vulva or cervix. Our case was present in the form of an ovarian mass. Therefore, an aggressive surgery was not performed. Since our patient was in the reproductive age, fertility-preserving surgery was performed. Our patient was then treated systemically by medical oncology. In conclusion, a rare malignancy group was investigated in the present study along with an evaluation of the current literature.
